# Commentary: APOE e4 Genotype Predicts Severe COVID-19 in the UK Biobank Community Cohort

**DOI:** 10.3389/fimmu.2020.01939

**Published:** 2020-09-15

**Authors:** Kyle Kasparian, David Graykowski, Eiron Cudaback

**Affiliations:** Department of Health Sciences, DePaul University, Chicago, IL, United States

**Keywords:** COVID-19, APOE and inflammation, APOE and COVID-19, APOE, APOE and SARS-CoV-2, APOE e4, APOE e4 and COVID-19, APOE e4 and inflammation

On March 11th, 2020, the World Health Organization declared COVID-19 a pandemic. This respiratory disease results from infection by the novel SARS-CoV-2 virus, and commonly presents with flu-like symptoms, including cough, fever, and fatigue. More severe cases, generally defined by hospitalization, intubation, and/or mortality, can also exhibit dyspnea, lymphopenia, and hypoalbuminemia with concomitant increases in the proinflammatory cytokines interleukin 6 (IL-6), tumor necrosis factor alpha (TNF-α), and interleukin 1 beta (IL-1ß) (1, 2). In addition, common COVID-19 comorbidities include dementia, heart disease, and hypertension (3–5). Clinical observations notwithstanding, surprisingly little is known about the molecular determinants that underlie this disease. Interestingly, the multiallelic apolipoprotein E gene (*APOE*), an important regulator of cholesterol homeostasis, has been similarly linked to Alzheimer's disease and cardiovascular disease (6–8).

Three common *APOE* alleles exist in the population, ε*2*, ε*3*, and ε*4*. To investigate the relative contribution of *APOE* genotype to the risk for developing severe COVID-19, Kuo et al. analyzed case data from the UK Biobank (UKB) cohort (9), a study of 322,948 subjects of European ancestry, with a mean age of 68 years. Subjects were sorted by *APOE* genotype, and the number of positive COVID-19 tests between March 16th and April 26th, 2020 was recorded for each group. The authors found that individuals homozygous for *APOE* ε*4* were more likely to test positive for COVID-19, and thus severe disease, compared the group homozygous for *APOE* ε*3* (9). Given the urgency for genetic determinants associated with COVID-19 severity, Kuo et al. continued their investigation with the addition of more test results and mortality data (10). The study confirmed their initial findings, additionally reaching genome-wide significance for the association of *APOE* ε*4*ε*4* genotype with COVID-19 test positivity. The initial analysis characterized positive cases as severe COVID-19 cases given positive tests were registered from March 16th to April 26th, 2020, a window of time in which testing was largely restricted to hospitalized patients. Importantly, however, the less restrictive temporal parameters established in the follow-up study significantly undermine this premise. Nevertheless, the mortality data presented (calculated by quantifying deaths after confirmed positive) suggests increased disease severity in these subjects (10). Moreover, they reported that *APOE* ε*4*ε*4* genotype was associated with a 4-fold increase in mortality after testing positive relative to *APOE* ε*3*ε*3* individuals. These data suggest that the *APOE* ε*4*ε*4* genotype represents a significant risk for the development of severe COVID-19, as well as death following infection. Interestingly, *APOE* ε*3*ε*4* COVID-19 positive subjects did not exhibit a significant difference in mortality compared to *APOE* ε*3*ε*3* positive subjects, and only a slight increase in positivity. This observation implies the absence of any gene dosage effects, underscoring the required inheritance of two ε*4* copies to acquire this genetic risk. Although these analyses identify *APOE* ε*4*ε*4* genotype as a potentially valuable genetic determinant for development of severe COVID-19, gaps remain in our current understanding of how specific mechanisms drive this association.

In addition to its critical function in lipid transport, *APOE* has also been shown to modulate important innate immune responses key to inflammation. For example, human subjects harboring *APOE* ε*4* exhibit significantly higher plasma levels of proinflammatory cytokines (IL 6 and TNFα) following IV challenge with the bacterial endotoxin, lipopolysaccharide (LPS) (11). A hallmark of COVID-19 infection is aberrant inflammation resulting from a dysfunctional immune response. Given the data from Kuo et al. illustrating a strong association between severe COVID-19 cases and *APOE* ε*4*ε*4* genotype independent of the comorbidities, it is plausible that *APOE* modulates COVID-19 disease severity by regulating pro-inflammatory pathways in a genotype dependent manner.

Recently, there has been increased concern regarding the potential for chronic neurodegenerative effects following COVID-19 infection (12–14). *APOE* ε*4* has also been shown to exacerbate inflammation in the CNS following stressors that mimic infection in the periphery. Peripheral administration of LPS resulted in increased expression of the proinflammatory chemokine CCL3 in the brains of *APOE* TR mice in a genotype dependent manner (ε*4* >ε*3*) (15). Furthermore, individuals carrying the *APOE* ε*4* allele exhibited accelerated breakdown of the blood brain barrier (16, 17). Thus, this not only creates concern for exacerbated inflammation in the periphery contributing to a cytokine storm during infection, yet also the potential for increased risk of long-term neurodegeneration in *APOE* ε*4* infected patients.

The present studies come at a time when the identification of genetic factors influencing the development of COVID-19 are essential. Kuo and colleagues help to shed light on the association between *APOE* ε*4* and severe COVID-19 infection, and establish a starting point from which to initiate empirical and replicable studies aimed at investigating the molecular nature of such genetic risks. Indeed, these findings encourage comprehensive interpretations of population-specific susceptibilities to COVID-19 like those observed among African Americans (18), and the role that variation in *APOE* allele frequency may play (19). Their work also provides an important foundation from which to explore the biological mechanisms that underlie these associations. Given the compelling evidence linking aberrant inflammation to severe COVID-19 (20), and the association of *APOE* ε*4* with exacerbated inflammation, future studies should aim to determine if *APOE* ε*4* is associated with increased inflammatory responses in the periphery following COVID-19 infection. This could easily be demonstrated by investigating plasma cytokine levels, as well as other immunological markers. Additionally, COVID-19 infection poses as a threat to the CNS given neuroinflammation is a universal characteristic across an array of neurodegenerative diseases. Therefore, longitudinal effects of COVID-19 infection in *APOE* ε*4* carriers, which exhibit exacerbated neuroinflammatory responses (21), may lead to exacerbated risk for developing these aliments. Taken together, future studies are necessary to elucidate an association between *APOE* ε*4*, inflammation, and COVID-19 infection, and characterize the biological mechanisms underlying this link. These studies should be strongly considered by the research community, because if an association exists, then millions of *APOE* ε*4* carriers worldwide must take additional precautions to prevent developing a devastating and life-threatening disease.

## Author Contributions

KK, DG, and EC conceived of and wrote the manuscript. All authors contributed to the article and approved the submitted version.

## Conflict of Interest

The authors declare that the research was conducted in the absence of any commercial or financial relationships that could be construed as a potential conflict of interest.
